# Genomic and transcriptomic analysis of MSI-H colorectal cancer patients with targetable alterations identifies clinical implications for immunotherapy

**DOI:** 10.3389/fimmu.2022.974793

**Published:** 2023-01-09

**Authors:** Hanju Hua, Wenguang He, Nan Chen, Yinjun He, Guosheng Wu, Feng Ye, Xile Zhou, Yandong Li, Yongfeng Ding, Weixiang Zhong, Lisong Teng, Weiqin Jiang, Qinsong Sheng

**Affiliations:** ^1^ Department of Colorectal Surgery, The First Affiliated Hospital, Zhejiang University, Hangzhou, China; ^2^ Department of Radiology, First Affiliated Hospital, Zhejiang University School of Medicine, Hangzhou, China; ^3^ Department of Colorectal Surgery, Yuyao Hospital of Traditional Chinese Medicine, Yuyao, China; ^4^ College of Medicine, Zhejiang University, Hangzhou, China; ^5^ Department of Medical Oncology, First Affiliated Hospital, College of Medicine, Zhejiang University, Hangzhou, China; ^6^ Department of Pathology, First Affiliated Hospital, College of Medicine, Zhejiang University, Hangzhou, China; ^7^ Department of Surgical Oncology, First Affiliated Hospital, College of Medicine, Zhejiang University, Hangzhou, China

**Keywords:** MSI-H cancer, targetable alterations, TME (tumor microenvironment), immunotherapy, GEP

## Abstract

**Introduction:**

Targetable alterations such as BRAFV600E mutation and NTRK fusion are enriched in microsatellite instability-high (MSI-H) colorectal cancer (CRC). MSI-H with targetable alterations (MSI-H altered) might present unique opportunities for both targeted therapy and immunotherapy. We systematically evaluated the molecular characteristics and immune-related features of MSI-H altered and MSI-H without targetable alterations (MSI-H wt) CRC patients in our study.

**Methods:**

Among 1938 continuously enrolled CRC patients, 126 patients with MSI-H status (6.50%) were included in this retrospective study. Genomic and transcriptomic data were investigated by next-generation sequencing (NGS) and gene expression profiling (GEP), respectively.

**Results:**

BRAFV600E, NTRK1, and FGFR2 mutations were the most frequent targetable alterations in MSI-H CRC patients. The MSI-H altered phenotype was significantly associated with older age (p< 0.001), right side (p=0.024) and females (p= 0.036). No lynch syndrome (LS) patients were identified in MSI-H altered group. The tumor mutational burden (TMB), and tumor neoantigen burden (TNB) of MSI-H altered and wt subgroups were comparable (p<0.05). Subsequently, transcriptomic study analysis further revealed MSI-H altered CRC patients were linked to an immune-active tumor microenvironment with higher levels of Teff IFN-gamma, CYT, and MERCK 18 signatures, and lower levels of the IPRES gene signature, EMT and TGF Beta signatures. In addition, case study supported MSI-H CRC patient harboring targetable alterations might also achieved a long-term disease-free survival benefit from immunotherapy.

**Discussion:**

Our study preliminary revealed MSI-H altered as a novel subtype of MSI-H CRC patients with unique molecular signatures and immune-active tumor microenvironment. Given the accessibility of immune checkpoint inhibitors (ICIs) treatment, our results might provide clinical evidence for immunotherapy in MSI-H CRC patients with targetable alterations.

## Introduction

*BRAF^V600E^*, *KRAS^G12C^* mutation, *ERBB2* (*HER2*) amplification, and various receptor tyrosine kinase (RTK) fusions are rare but potentially therapeutically relevant in colorectal cancer (CRC) (1, 2). Targetable variations such as *NTRK* fusion and *BRAF^V600E^* mutation were reported to be enriched in microsatellite instability-high (MSI-H)/deficiencies in DNA mismatch repair (dMMR) CRC tumors (3, 4). With the inhibitors of these powerful oncogenic drivers being approved by the Food and Drug Administration (FDA) in solid tumors, there has been an expanding list of targeted therapy options for patients with metastatic colorectal cancer (mCRC) (5–7). The KEYNOTE-177 phase III study presented immune checkpoint inhibitors (ICIs; pembrolizumab) led to a prolonged progression-free survival (PFS) than chemotherapy when received as the first-line therapy for MSI-H/dMMR mCRC patients (8). However, for MSI-H CRC patients with targetable alterations (MSI-H altered), either targeted therapy or immunotherapy to be given to achieve the optimal clinical benefits that deserve further consideration.

In lung cancer, a study presented tyrosine kinase inhibitor (TKI) treatment naïve, programmed death ligand-1 (PD-L1) positive, *EGFR*-mutant positive patients could barely benefit from pembrolizumab (9, 10). As MSI-H CRC patients with targetable alterations are rare and poorly characterized, it is still controversial regarding the optimized clinical options for available treatments including targeted therapy and immunotherapy. It was reported that larotrectinib (receptor tyrosine kinases [RTK] inhibitor of *NTRK* fusion) in gastrointestinal (GI) cancer confirmed its response and ability of disease control in heavily pretreated GI patients (7 MSI-H and 1 microsatellite stable tumors [MSS] CRC patients), and demonstrated 4 of 8 CRC patients achieved partial response (11). Another study reported that MSI-H CRC patients harboring *NTRK* gene rearrangements had a durable response to targeted therapy, but not to immunotherapy (12). In contrast, two *NTRK* fusion-positive MSI-H cases received immunotherapy and achieved complete response (CR) as the best overall response (more than 3.5 years disease-free survival, DFS) and stable disease (SD), respectively (13). Similarly, the KEYNOTE-177 study also observed that patients with *BRAF^V600E^* mutant and those with wild-type MSI-H tumors benefit equally from immunotherapy with PD-1 blockade treatment. Taken together, there is still no consensus on the priority of targeted therapy or immunotherapy for patients with MSI-H altered, optimal treatment strategies need to be further explored.

Currently, comprehensive molecular and functional analysis to address whether these targetable variations confer oncogene addiction or immune environment, or to suggest perspectives on the treatment options were unavailable. We therefore systematically analyzed the molecular landscape of patients with MSI-H CRC with/without targetable alterations, in regard to investigating the prevalence of genetic alterations, co-occurrence with relevant biomarkers/oncogenic drivers, and immunotherapy-related markers.

## Methods

### Patients

We developed a cohort of patients diagnosed with CRC from the First Affiliated Hospital of Zhejiang University. The entire cohort was annotated for clinicopathological details including stage, grade and targetable alterations mutation status. *BRAF^V600E^, KRAS^G12C^*, *HER-2* amplification and 16 reported *RTK* fusions were defined as targetable alterations, as they have been reported in the CRC and their corresponding inhibitors have been approved by the FDA (**Supplementary Table 1**). Clinical data and medical records were retrieved from patients’ medical records. This study was approved by the research ethics committee of the First Affiliated Hospital, Zhejiang University School of Medicine, China (NO. IIT20210185B). And written informed consents were obtained from all the patients.

### Data acquisition

Genome Atlas-Colorectal Cancer (TCGA)- colon adenocarcinoma (COAD)/rectal adenocarcinoma (READ) cohorts with DNA methylation profiles, and mutational data were downloaded (https://portal.gdc.cancer.gov) and analyzed in the present study (14). The average methylation level of four cytosine-phosphate-guanine (CpG) sites on *MLH1* gene (cg00893636, cg21490561, cg11600697, and cg23658326) was used to represent *MLH1* methylation (15).

### DNA sequencing and data analysis

Tissue samples were sequenced using a panel targeting 520 cancer-related genes (Burning Rock, Guangzhou China). DNA isolation and targeted sequencing were performed in Burning Rock Biotech, a commercial clinical laboratory accredited by the College of American Pathologist (CAP) and certified by the Clinical Laboratory Improvement Amendments (CLIA). Genomic DNA was extracted from formalin-fixed, paraffin-embedded (FFPE) samples using QIAamp DNA FFPE tissue kit (Qiagen, Hilden, Germany). Fragments between 200–400bp from the sheared tissue DNA were purified (Agencourt AMPure XP Kit, Beckman Coulter, CA, USA), hybridized, and amplified. Target capture was performed using a commercial panel consisting of 520 cancer related genes, spanning 1.64 megabases of the human genome. Sequence data were mapped to the reference human genome 19 using Burrows-Wheeler Aligner version 0.7.10. Local alignment optimization, duplication marking and variant calling were performed using Genome Analysis Tool Kit version 3.2, and VarScan version 2.4.3. Tissue samples were compared against their own white blood cells control to identify somatic variants. Structural rearrangement was analyzed using an in-house algorithm markSV.

The MSI status of tissue samples were determined based on a read-count-distribution-based method as previously published (16, 17). Tumor mutational burden (TMB) per patient was computed as a ratio between the total number of non-synonymous mutations detected and the total coding region size of the panel. Tumor neoantigen burden (TNB) was calculated as the total number of all mutations which may generate neoantigens per megabase. MSI status, TMB, and TNB were all calculated by Burning Rock 520 cancer-related gene panel (Guangzhou, China).

### RNA sequencing and data analysis

RNA was also isolated from FFPE samples using an AllPrep DNA/RNA FFPE Kit (Qiagen, Hilden, Germany). The quantity and quality of extracted RNA was quantified by Qubit RNA HS assay (Thermo Fisher Scientific, Waltham, MA, USA) and LabChip GXII touch 24 (Perkin Elmer, Waltham, MA, USA), respectively. Fragmented RNA was subjected to strand-specific cDNA synthesis, followed by dA-tailing, unique molecular identifier (UMI) adaptor ligation, PCR amplification, and hybridization with capture probe baits of the Gene expression profiling (GEP) panel (Burning Rock Biotech, Guangzhou, China). The GEP platform is a unique 218-gene expression panel that quantifies 83 immune-related genes in human solid cancers (**Supplementary Table 3**). The prepared NGS libraries were sequenced on a NovaSeq 6000 system (Illumina, Inc., San Diego, CA, USA). A threshold of >25 million reads per sample was set. After deduplication and removing UMI from the sequence header, adaptors, and low-quality reads were removed. The cleaned reads were aligned to the human reference genome 19 by STAR (2.7.3a)., then the consensus reads were created using homebrew software based on UMI sequence and read alignment position. Consensus reads were aligned again to the human reference genome 19 by STAR2 (2.7.3a). To search gene fusion in the transcriptome, STAR-Fusion (v1.8.1) was applied to chimeric-junction files generated in the previous STAR alignment to identify fusions.

Immune-related scores such as T effector (Teff) interferon-gamma (IFN-gamma), the cytolytic (CYT) activity (18), MERK 18 signature (19), innate PD-1 resistance (IPRES) gene signature (20), epithelial–mesenchymal transition (EMT) (21, 22) and transforming growth factor β (TGFβ) (23) were analyzed using GEP data.

### Methylation-specific polymerase chain reaction (MSP)

We followed a well-established method to analyze MSP (24).

**Table:** 

MLH1 methylated alleles (Forward)	5′-AACGAATT AATAGGAAGAGCGGATAGCG-3′
MLH1 methylated alleles (Reverse)	5′-CGTCCCTCCC TAAAACGACTACCC-3′
MLH1 unmethylated alleles (Forward)	5′- TAAAAATGAATTAATAGGAAGAGTGGATAGTG-3′
MLH1 unmethylated alleles (Reverse)	5′-AATCTCTTCATCCCTCCCTAAAACA-3′

PCR conditions for *MLH1* methylated and *MLH1* unmethylated primers were initial denaturation at 95°C for 10 minutes, 37 cycles of 30 seconds denaturation at 95°C, 45 seconds annealing at 55°C and 30 seconds extension at 72°C. Then, the products were stored at 4°C.

### Immunohistochemistry (IHC)

IHC staining of FFEP tumor tissue sections (4μm thick) was performed to examine the expression of PD-L1 by 22C3 pharmDx (Dako, Carpinteria, CA), based on tumor proportion score (TPS), and combined positive score (CPS) (25). Combined positivity score (CPS) is defined as the percentage of total PD-L1+ cells (tumor cells and immune cells) divided by the total number of tumor cells. Tumor proportion score (TPS) is defined as the percentage of tumor cells with membranous PD-L1 expression. PC is the positive control; NC is the negative control.

### Statistics

All the data were analyzed using the R package (R version 4.0.2; R: The R-Project for Statistical Computing, Vienna, Austria). The differential expression genes (DEGs) were preliminarily screened with “limma” R package. Genes with adjusted P < 0.05 were identified as DEGs. Gene Ontology (GO) analysis revealed the signaling pathways associated with these DEGs. KEGG analysis was performed on the DEGs between MSI-H altered and MSI-H wt groups, based on the GSEA software (https://www.gsea-msigdb.org/gsea/login.jsp). Results with normalized enrichment score (NES) < − 1.5, and P. adjust < 0.05 were considered to be significantly enriched.

The Spearman rank correlation coefficient was used to measure the relationship between two variables. The Wilcoxon rank sum test and the Wilcoxon matched-pairs signed rank sum test with Bonferroni correction were used to compare the difference between two or more sets of quantitative data. All P-values were two-sided, and P-values less than 0.05 were considered statistically significant.

## Result

### Study population

**Figure 1:**
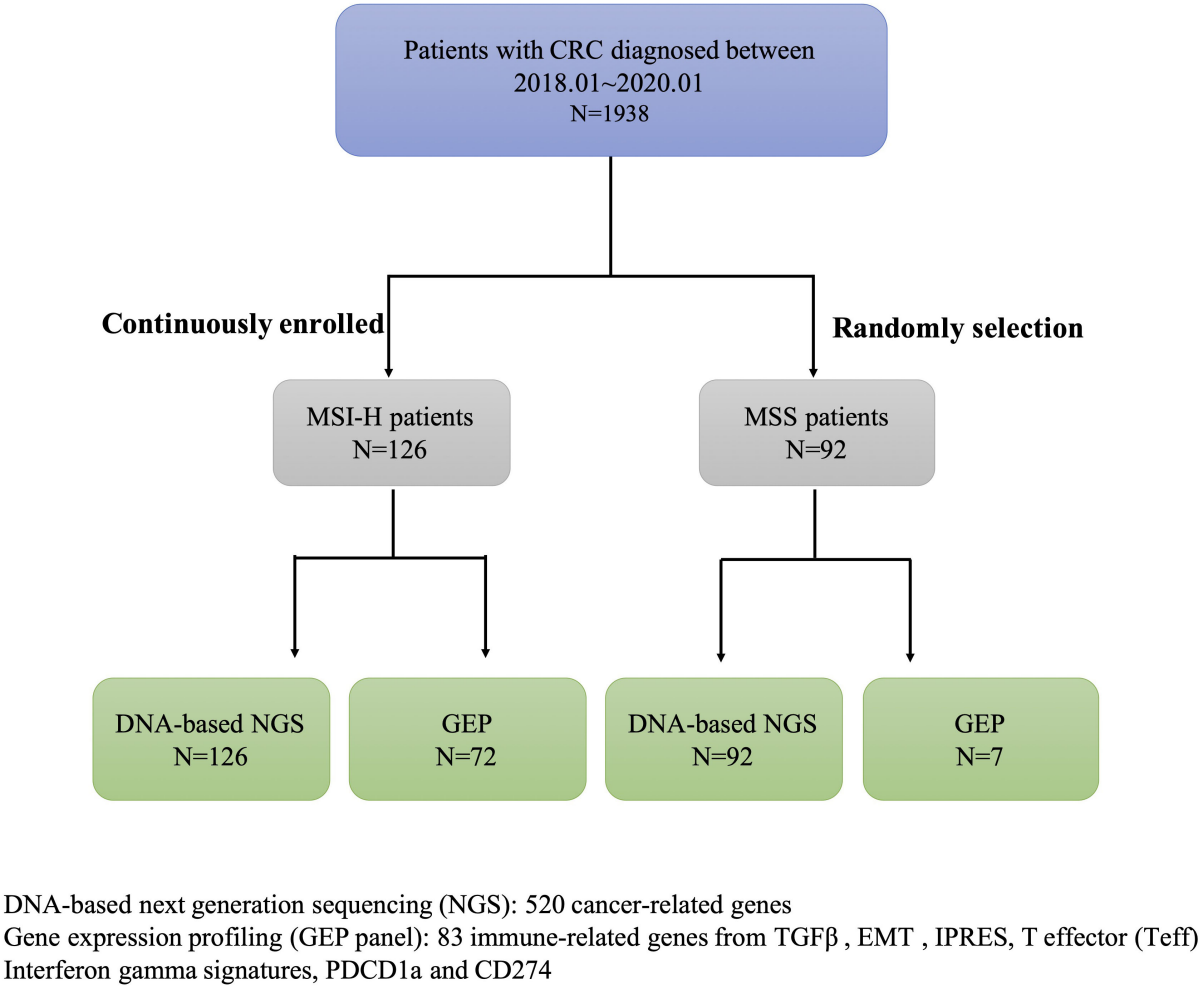
Detailed study design and patient selection.

In this study, we screened the MSI status in 1938 CRC patients diagnosed between 01/01/2018 and 01/01/2020 from the first affiliated hospital of Zhejiang University, collected clinical data and pedigree information, and finally identified 126 CRC patients with MSI-H. This study also included 99 CRC patients with MSS (**Supplementary Table 1**). DNA-based next-generation sequencing (NGS) of 520 tumor-related genes was conducted in all samples. GEP panel covering 83 immune-related genes (from four immune signatures and two checkpoint genes *PDCD1*, *CD274*) was conducted in 72/126 MSI-H samples and 7/96 MSS samples (**Figure 1**; **Supplementary Table 2**). A higher TMB was associated with MSI-H patients (**Supplementary Table 1**). **Supplementary Figure 1** showed the mutational landscape of gene mutations, indicating that MSI-H had a higher frequency of mutations than MSS.

### Genomic features of MSI-H altered and MSI-H wt subgroups

We further analyzed the public cohort (TCGA-COAD/READ databases) to investigate mutation status in MSI-H and MSS CRC patients. We found *BRAF^V600E^* mutation and *RTK* fusions enriched in MSI-H CRC patients, but *KRAS^G12C^* mutation and *ERBB2* (*HER2*) amplification only occurred in MSS CRC patients (**Supplementary Figure 2**). MSI-H was detected in 6.50% (126/1938) of the continuously enrolled CRC patients in our center, and targetable mutations were detected in (30/126) of the MSI-H patients, *BRAF ^V600E^*, *NTRK1* and *FGFR2* fusion were the most frequent targetable alterations in this group. And still no *KRAS^G12C^* mutation and *ERBB2* (*HER2*) amplification were captured (**Supplementary Figure 2**). There was no significant difference in the tumor stage (p = 0.838), the TMB (p = 0.074), the TNB (p = 0.066), the cytokeratin 7 (CK7, p = 0.602), and the CK 20 (p = 0.189) of CRC patients in the MSI-H altered and MSI-H wt subgroups (**Table 1**). MSI-H with targetable alterations were more often in older patients (p < 0.001), and right side (p=0.024). In addition, we also found MSI-H altered CRC patients were more often in women (60%, 18/30), while MSI-H wt were frequent in men (62.5%, 60/96).

**Table 1:** Clinicopathological characteristics of the MSI-H CRC patients with and without targetable alterations in this study.

	Overall (n = 126)	MSI-H altered (n = 30)	MSI-H wt (n = 96)	p
**Age**				
Mean (SD)	59.98 (13.61)	71.69 (10.43)	56.44 (12.46)	<0.001
Median [IQR]	59.00 [52.00, 70.00]	73.00 [64.00, 80.00]	55.00 [48.75, 68.00]	
Missing	1 (0.8)	1 (3.3)	0 (0.0)	
**Gender**				
female	54 (42.9)	18 (60.0)	36 (37.5)	0.036
male	72 (57.1)	12 (40.0)	60 (62.5)	
**Stage**				
I	8 (6.3)	2 (6.7)	6 (6.2)	0.838
II	63 (50.0)	13 (43.3)	50 (52.1)	
III	36 (28.6)	10 (33.3)	26 (27.1)	
IV	19 (15.1)	5 (16.7)	14 (14.6)	
**Tumor location**				
Right	60 (47.6)	20 (66.7)	40 (41.7)	0.024
Left	28 (22.2)	2 (6.7)	26 (27.1)	
NA	38 (30.2)	8 (26.7)	30 (31.2)	
**TMB**				
Mean (SD)	75.05 (60.65)	62.41 (49.64)	79.00 (63.42)	0.074
Median [IQR]	58.82 [41.87, 78.26]	50.35 [40.13, 69.79]	61.31 [44.56, 86.49]	
**TNB**				
Mean (SD)	72.92 (55.60)	62.00 (51.91)	76.09 (56.49)	0.066
Median [IQR]	61.00 [36.00, 93.50]	51.00 [29.50, 62.00]	71.00 [36.00, 97.00]	
Missing	6 (4.8)	3 (10.0)	3 (3.1)	
**CK7**				
-	34 (27.0)	10 (33.3)	24 (25.0)	0.602
+	11 (8.7)	3 (10.0)	8 (8.3)	
NA	81 (64.3)	17 (56.7)	64 (66.7)	
**CK20**				
-	3 (2.4)	2 (6.7)	1 (1.0)	0.186
+	52 (41.3)	13 (43.3)	39 (40.6)	
NA	71 (56.3)	15 (50.0)	56 (58.3)	

TMB, tumor mutational burden; TNB, tumor neoantigen burden; CK, cytokeratin; NA, not available.

MSI-H altered: MSI-H with targetable alteration, MSI-H wt: MSI-H without targetable alteration.

**Figure 2:**
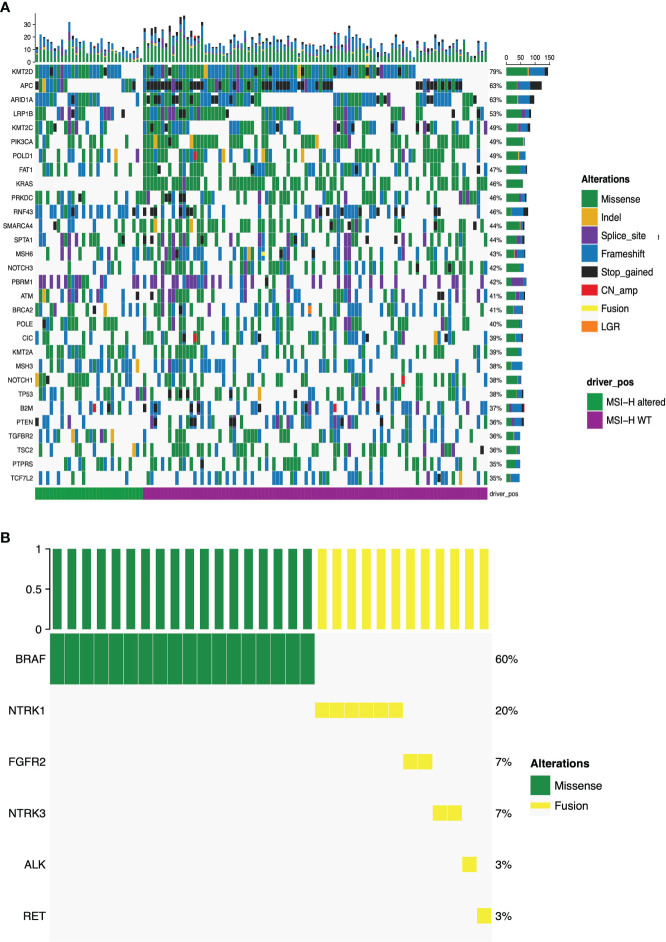
Mutational landscape of the MSI-H altered and wt groups. **(A)** The prevalence of major targetable alterations in MSI-H altered and wt groups; **(B)** The prevalence of *BRAF* missense and other fusions in MSI-H.

**Figure 3:**
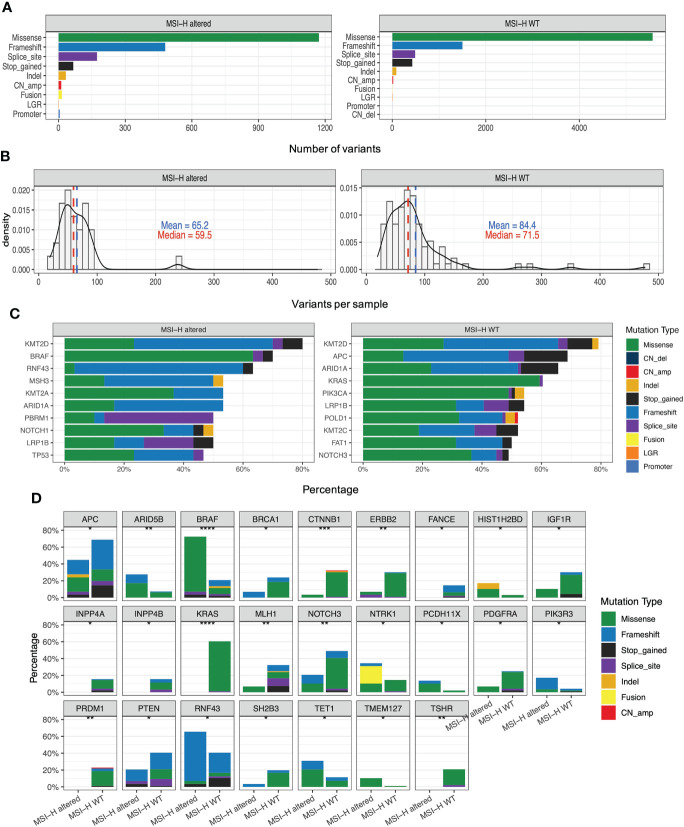
Genomic analysis in the MSI-H altered and wt groups. **(A)** Distribution of the variant types in the MSI-H altered and wt groups; **(B)** Number of variants per sample in MSI-H altered and wt groups. **(C)** Top 10 frequently mutated genes in tumors from MSI-H altered (left) and wt (right). **(D)** Mutation frequency of several CRC-related key genes in MSI-H altered and wt groups, respectively.

**Figure 2A** presented the mutational landscape of gene mutations indicating that the MSI-H altered group had a higher frequency of mutations than the MSI-H wt group. And *BRAF* missense (V600E) and other targetable alterations (fusion) were mutually exclusive (**Figures 2A** **, B**). Regarding the distinctions of tumor targetable variants between the two subgroups, the distribution of variant types and the number of variants per sample of MSI-H altered and MSI-H wt were displayed (**Figures 3A** **, B**). Furthermore, the top 10 frequently mutated genes from MSI-H altered and MSI-H wt were shown in **Figure 3C**. More specifically, *KRAS*, *CTNNB1, ERBB2* and *APC* gene variants occurred more frequently in MSI-H wt patients in our cohort (**Figure 3D**). We further explored the difference in molecular mechanisms between MSI-H altered (n=29) and MSI-H WT (n=21) groups through GSEA. There are 128 gene sets were significantly enriched in the MSI-H altered group, including 30 gene sets were significantly enriched (p<0.05). Most of the enriched pathways were cancer-related, including the CRC-related pathway and WNT, VEGF, TGF_BETA signaling pathways (**Supplementary Figure 3**).

### MMR genes in MSI-H altered and MSI-H wt subgroups

**Figure 4:**
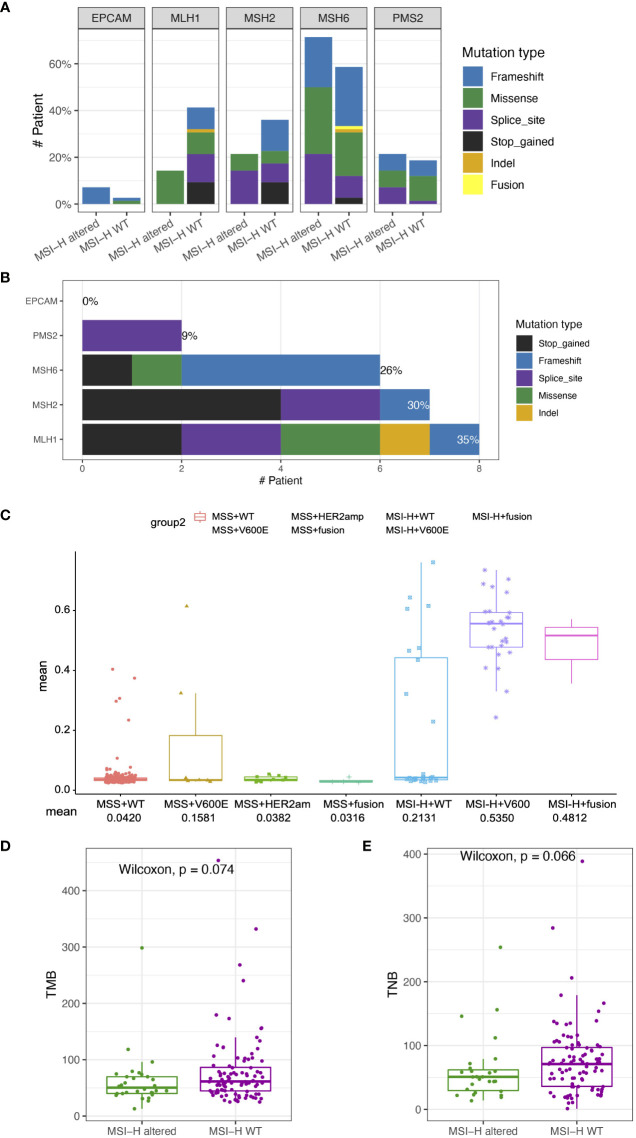
Somatic variant features of tumor from MSI-H altered and wt groups **(A)** Distribution and types of somatic variants in MSI-H altered and wt groups. **(B)** Distribution and types of genetic variants in patients with Lynch syndrome. **(C)** Comparison of *MLH1* methylation levels among the MSS wt, MSS *BRAF* ^V600E^, MSS *Her2* amplification, MSS fusion, MSI-H wt, MSI-H *BRAF^V600E^*, MSI-H fusion groups. **(D, E)** The TMB and TNB scores of CRC patients in the MSI-H altered and wt subgroups. The analyzed data of **(C)** was obtained from The Cancer Genome Atlas (TCGA) database.

Lynch syndrome (LS) is induced by carrying inherited pathogenic or likely pathogenic (P/LP) variants in any of the five MMR genes, impairing the DNA MMR system (26). Distribution and types of somatic variants of *MLH1*, *MSH2*, *MSH6*, *PMS2* and *EPCAM* in 126 enrolled MSI-H CRC patients were presented in **Figures 4A, B**. Significantly high levels of somatic *MLH1* and *MSH2* variants were associated with the MSI-H wt group (**Figure 3A**). We also found the prevalence of patients with LS was 24% (23/96) in the MSI-H wt cohort. In contrast, there are no patients with LS in the MSI-H altered group, suggesting MMR germline mutations and targetable alterations were mutually exclusive. Among those 23 patients diagnosed with LS, about 35%, 30%, 26%, and 9% cases carried germline *MLH1*, *MSH2*, *MSH6*, and *PMS2* variants, respectively. And none of the *EPCAM*-related LS was diagnosed (**Figure 4B**).

Except for germline genetic aberrations, promoter methylation also could lead to loss of MMR protein expression, and induced sporadic CRC. We further analyzed the TCGA-COAD/READ databases to investigate the methylation status of *MLH1p* in the MSI-H and MSS CRC patients. The average methylation level of four cytosine phosphate guanine (CpG) sites on *MLH1* gene was used to represent the *MLH1p* methylation level. *MLH1p* methylation level of the MSI-H fusion and MSI-H *BRAF^V600E^* were significantly higher than that of MSS and MSI-H wt subgroups (**Figure 4C**), suggesting MSI-H altered patients tended to harbor genetic aberrations leading to the sporadic CRC, rather than the germline or somatic MMR gene aberrations.

### TMB/TNB in MSI-H altered and MSI-H wt subgroups

In addition to gene mutations related to targeted therapy, we also investigated the TMB, and TNB status that are closely associated with immunotherapy response. The median TMB of the MSI-H cohort was 58.82 (range: 41.87, 78.26) mutations/Mb. We conducted further subgroup analysis based on driver gene alteration and found that the TMB of the MSI-H altered group was numerically similar to that observed in the MSI-H wt group (p = 0.074; **Figure 4D**). In addition, no TMB difference was observed in the MSI-H subgroups, including the MSI-H *BRAF^V600E^*, the MSI-H fusion, and the MSI-H wt (p > 0.05, **Supplementary Figures 4A, B**). The TNB is directly used for neoantigen evaluation and may be considered an improved biomarker for immunotherapy compared with the TMB. We conducted the subgroup analysis based on driver gene alterations and found that the TNB of the MSI-H wt group was also similar as that in MSI-H altered group (p = 0.066; **Figure 3D**). The TNB of MSI-H wt group was statistically higher than that in the MSI-H fusion group (p = 0. 046; **Supplementary Figures 4C, D**).

### Gene expression signatures of MSI-H CRC MSI-H altered and MSI-H wt subgroups

Although the detection of targetable mutations by DNA-based sequencing provides evidence of the targeted therapy for MSI-H CRC patients, there is still no consensus on the clinical priority of targeted treatment or immunotherapy for these patients. In order to assess the benefits of immunotherapy in MSI-H patients with targetable alteration, the transcriptome data of 72 MSI-H samples were assessed using a 218-gene panel RNA-based sequencing platform, including 83 immune-related genes that fall into essential signaling pathways (**Supplementary Table 3**). In this study, RNA-based sequencing recovered one MSI-H patient with *NCOA4-RET* fusion that appeared driver negative by DNA-based sequencing, indicating a more comprehensive detection of fusions by the additional transcriptomic analysis.

**Figure 5:**
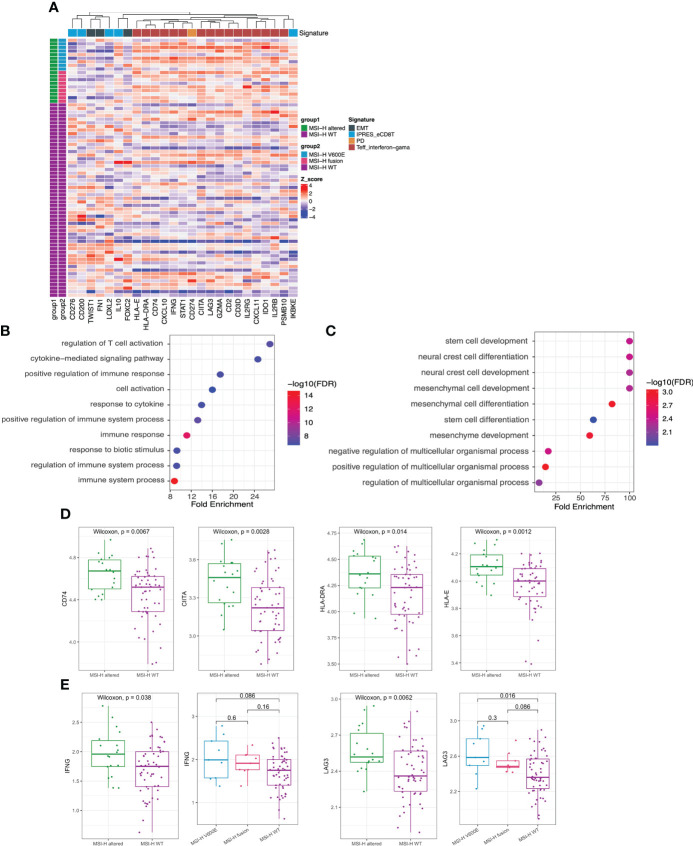
Transcriptomic analysis in MSI-H altered and wt groups. **(A)** 25 differentially expressed genes were identified by GEP (p<0.05). **(B, C)** GO function analysis of upregulated genes **(B)** and downregulated genes **(C)**. **(D, E)** Comparison of HLA-related genes **(D)**, representative immune-related *IFNG* and *LAG3* genes **(E)** in MSI-H altered and wt groups.

GEP panel detected 25 DEGs (p value < 0.05) when comparing MSI-H altered and MSI-H wt subgroups (**Figure 5A**). 6 DEGs were lower in the MSI-H altered group than those in MSI-H wt group. GO function analysis revealed that up-regulated DEGs were enriched in the T cell activation, cytokine mediate signaling pathways and positive regulation of immune response, while down-regulated DEGs were enriched in stem cell development, neural crest cell differentiation and development, indicating the differences in biological process between these two subgroups (**Figures 5B** **, C**).

We also observed a general pattern that the high expression levels across the immune-related genes in MSI-H altered patients. Notably, some human leukocyte antigen (HLA)-related genes (*CD74*, *CITA*, *HLA-DRA*, *HLA-E*), cytokine interferon gamma (*IFNG*) and immunomodulatory factor (*LAG3*) exhibited significantly higher enrichment levels in MSI-H-altered patients (**Figures 5D, E**; **Supplementary Figure 5A**). Taken together, distinctive features of biological processes and immune-related genes were found between MSI-H altered and MSI-H wt subgroups.

**Figure 6:**
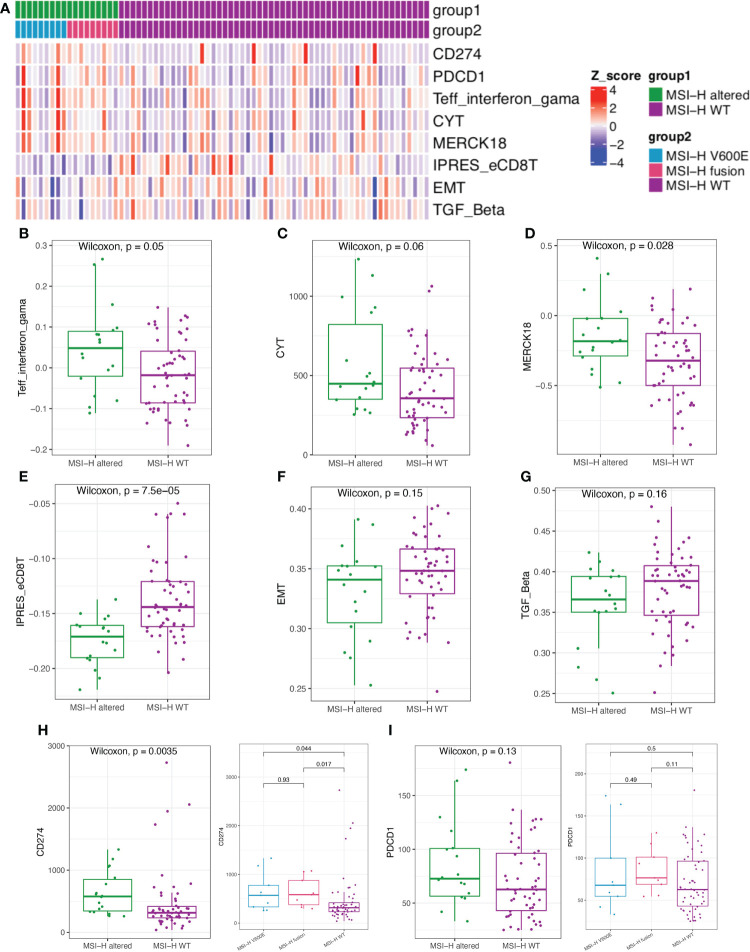
Comparison of the biomarkers for immune checkpoint inhibitors (ICIs) therapy between MSI-H altered and wt groups. **(A)** Heatmap of immune related scores in MSI-H altered and wt groups. **(B–D)** The boxplot indicated Teff IFN-gamma, CYT, and MERK 18 signature were reduced in MSI-H wt group. **(E–G)** The boxplot indicated the higher innate PD-1 resistance (IPRES) gene signature, EMT and TGF Beta in MSI-H wt group. **(H, I)** The boxplot indicated the higher the expression of immune checkpoints CD274 and PDCD1 in MSI-H altered group.

We further estimated the CYT activity, Teff IFN-gamma, MERK 18 signature, IPRES gene signature, EMT and TGFβ in MSI-H altered and MSI-H wt CRC patients (**Figure 6A**). Teff IFN-gamma, CYT, and MERK 18 signatures for prediction of clinical response to PD-1 checkpoint blockade were elevated in the MSI-H *BRAF^V600E^* and fusion groups (**Figures 6B–D**; **Supplementary Figure 5B**). The IPRES, EMT and TGFβ signatures for the prediction of resistance to PD-L1 or CTLA-4 blockade were elevated in the MSI-H wt group (**Figures 6E–G**; **Supplementary Figure 5B**). Clinical factors (age and sex) showed significant differences between the two compared groups. We further reviewed both these cofactors were significantly correlated with immune signatures (**Supplementary Figure 6**). After adjustment, the immune signature changes between the two compared groups were similar as before (**Supplementary Table 4**). In this study, we also analyzed the association of MSI-H status with some representative inhibitory checkpoint molecules. As expected, the expression of immune checkpoints *CD274* and *PDCD1* were significantly enriched in MSI-H altered group (**Figures 6H** **, I**). Taken together, these data suggested that MSI-H altered group tended to show an immune-active microenvironment, indicating that these patients might potentially benefit from immunotherapy.

### Clinical response of an immune checkpoint inhibitor (ICI)-treated MSI-H CRC patient

**Figure 7:**
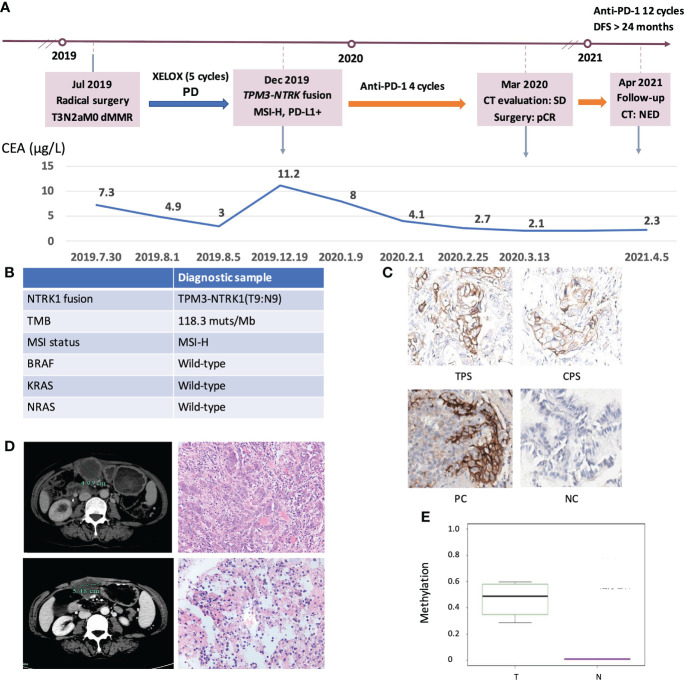
Clinical response of an ICIs-treated *NRKT* fusion MSI-H CRC patient. **(A)** Diagram of the course of disease management, showing different treatment regimens prescribed. **(B)** The molecular diagnosis of this case. **(C)** IHC staining of PD-L1 expressions in tumor proportion score (TPS), combined positive score (CPS), positive control (PC), and negative control (NC). **(D)** CT evaluation and HE images of the patient at the first (upper) and second (lower) surgical timepoint. **(E)** Comparison of *MLH1* methylation levels between *NRKT* fusion MSI-H tumor tissue and non-tumor tissue. T, tumor tissue; N, non-tumor tissue.

Finally, we explored the clinical impact of targetable mutations in the MSI-H CRC setting. We presented an MSI-H CRC case harboring *NTRK1* fusion who received anti-PD-1 treatment and achieved a durable response (**Figure 7A**). A 52-year-old woman presented to our hospital with an ascending colon mass and received radical resection in July 2019. The patient was diagnosed with CRC (pT3N2aM0, dMMR). After 5 cycles of XELOX, the patient’s disease progressed as assessed by CT imaging which indicated abdominal metastasis. NGS testing of the surgical tissue revealed *TPM3-NTRK1* (T9:N9, **Supplementary Figure 6**) fusion and the MSI-H phenotype, with no germline or somatic aberration in MMR genes (**Figure 7B**). The expression of PD-L1 was detected by IHC staining (**Figure 7C**). The patient started to receive anti-PD-1 monotherapy in December 2019. The patient was assessed as stable disease (SD) with an increase in tumor size based on the Response Evaluation Criteria on Solid Tumors (RECIST) version 1.1 after 4 cycles but with decreasing tumor serum marker carcinoembryonic antigen (CEA). The patient underwent surgical resection in March 2020. The postoperative pathological specimens showed extensive granulomatous inflammation with tissue necrosis, multinucleated giant cells, and no tumor cells observed, which was evaluated as pathological complete response (pCR) (**Figure 7D**). Postoperatively, the patient received another 12 cycles of PD-1 inhibitor monotherapy. The patient was assessed as having no evidence of disease (NED) till now and reached a disease-free survival (DFS) for more than 24 months. The methylation status of *MLH1p* in the tumor tissue of this case and the corresponding adjacent tissue were tested (**Figure 7E**). Taken together, the long DFS of this patient suggested that MSI-H CRC patients harboring targetable mutations might benefit from immunotherapy.

## Discussion

Immunotherapy has become the standard first-line treatment of patients with dMMR/MSI-H mCRC, however, not all MSI-H CRC patients benefit from anti-PD-1, reported objective response rate (ORR) was about 40% (27, 28), suggesting the heterogeneity in patients with MSI-H. In this study, we proposed that CRC patients with MSI-H and targetable alterations might be a novel CRC MSI-H subtype with unique clinical and molecular features based on comprehensive genomic and transcriptomic analysis, and would fill the clinical need to identify new targets and therapies for these patients.

An integrated genomic analysis of this MSI-H CRC cohort identified several important findings. Specifically, we observed a striking enrichment in *BRAF^V600E^* and *RTK* fusions in MSI-H CRC patients, while *KRAS^G12C^* mutation and *ERBB2* amplification only occurred in MSS CRC patients, probably due to the mutual exclusion between members of mitogen-activated protein kinase (MAPK) family. Furthermore, nearly all the *KRAS* missenses were associated with MSI-H wt patients in our cohort. It was reported that *BRAF* mutational status did not affect by ICIs treatment, but *KRAS* mutational status might have negative effects on ICIs treatment (27, 29, 30). Similarly, a study presented that tumors containing hot-spot mutations in *RAS* genes didn’t have a PFS benefit from the PD-1 blockade therapy (8). And the objective response of nivolumab monotherapy in patients with *KRAS* mutations (7/26; 27%) was significantly lower than in patients without *KRAS* and *BRAF* mutations (12/29; 41%) (28). These findings potentially indicated that MSI-H altered patients might benefit from immunotherapy.

Here we showed that targetable alterations identify a new MSI-H CRC molecular subtype with specific clinical and molecular features. The investigated targetable alterations were more frequent in elderly MSI-H CRC patients. It might be reflective of the exclusion of patients with LS who are generally diagnosed at an earlier age or the younger age of patients enrolled (18, 31). It was reported that the expression of CK7 was associated with MLH-1 and p53 expression, and also with the microsatellite status, *BRAF*, and *KRA*S pattern (32, 33). In addition, CK20 was not expressed in MSI-H cases (32). However, we observed 3 MSI-H altered cases expressed CK7 protein, and 13 (43.3%) MSI-H altered tumors expressed CK20 protein. Moreover, there is no difference in expression of these two immunohistochemical markers (CK7 and CK20) between these two entities, suggesting CK7 and CK20 markers could not differentiate MSI-H wt and MSI-altered groups.

*MLH1* promoter methylation is one of the major mechanisms for sporadic MSI-H CRC tumors (34). Our data showed a significant difference in *MLH1* promoter methylation between the tumor and adjacent tissue of an MSI-H CRC patient with *TPM3-NTRK1* fusion. Further public database analyses showed that *MLH1* promoter methylation between MSI-H fusion and MSI-H *BRAF^V600E^* subgroups was similar, while significant differences were found between both these two subgroups and MSI-H wt group. As previously reported, CRC patients containing *BRAF^V600E^* had a CpG island methylator phenotype (CIMP) characterized by aberrant hypermethylation of *MLH1* promoter (35). *MLH1* gene silencing resulted in MSI-H and a hypermutable phenotype. *BRAF^V600E^* mutations were associated with sporadic dMMR/MSI-H CRC and were rarely reported in patients with LS (36). The results presented here, in conjunction with previous studies, suggested that targetable alterations in the MSI-H population may share the same mechanism.

Consistent with previous research, our study observed a higher TMB associated with MSI-H compared with MSS in patients with CRC (37). TMB appears to be an important independent biomarker in solid tumors to stratify patients for likelihood of response to the ICIs therapy (38). TMB generates neoantigens and causes tumor immunogenicity. TNB is also directly used for neoantigen evaluation and could be considered an improved biomarker for the ICIs therapy compared with the TMB (39). High TNB produces neoantigens, contributing to an inflammatory microenvironment, which ultimately leads to improved outcomes following the ICIs therapy (40, 41). However, both TMB and TNB have no significant difference between MSI-H altered and MSI-H wt subgroups, which might imply that genomic-based analysis alone is difficult to guide the selection of immunotherapy and targeted therapy in MSI-H altered patients, suggesting in-depth exploration is required.

To better characterize the immune characteristics of MSI-H altered CRC patients, transcriptomic data were addressed by the GEP platform. Our results further revealed that the immune-active signatures were enriched in MSI-H altered patients, while the immune-suppressive signatures were accumulated in MSI-H wt patients. In addition, significantly higher expression levels of immune regulatory molecules like PD-1 and PD-L1 were observed in MSI-H altered patients. Immune-related molecules such as *IFNG* and *LAG3* are also enriched in MSI-H altered tumors, suggesting that increased anti–PD-1/PD-L1 efficacy in this class of tumors might be observed when combined with immune modulators targeting other suppressive pathways (42–44). Taken together, DEGs and immune related signatures identified in MSI-H altered CRC patients suggested that the upregulation of multiple pro-inflammatory signals accompanied by the expression of PD-1 and PD-L1 contribute to an immune “active” tumor microenvironment that can be successfully modulated by the ICIs therapy.

In fact, in many solid tumors, targeted therapy is the priority for patients with targetable alteration (45, 46). Studies showed NSCLC patients with powerful driver mutations could barely benefit from ICIs treatment. Recently, a similar study also showed that the MSI-H CRC harboring *NTRK* gene rearrangements had a durable response to targeted therapy but not to immunotherapy (4). However, the benefit from targeted strategies might be transient because acquired resistance is inevitable and difficult to overcome. In addition, the safety profiles and financial burden of targetable therapies should also be taken into account in clinical decisions. In this study, we presented a case of MSI-H CRC patient with *NTRK1* fusion who received anti-PD-1 treatment and experienced an increase in tumor size (SD) but pCR after resection. In conjunction with previous research, we need to consider the possibility of pseudo-progression induced by the ICIs therapy (47, 48). Although the underlying mechanism of this phenomenon is still undefined, it is a noted phenomenon of the ICIs treatment and might potentially influence clinical decisions. Therefore, a comprehensive evaluation including radiological surveillance, serum tumor markers (CEA, CA199 and CA125) monitoring, pathological examination, and molecular diagnosis based on genomic and transcriptomic analyses would help identify the potential population that might benefit from immunotherapy.

Despite these findings, there are some limitations to this observation study. Firstly, the lack of pretreatment and follow-up information, and drug sensitivity for the MSI-H CRC cohort from both in-house and TCGA cohort and the relevant *in vivo* experiments did not allow us to directly prove our hypothesis in this study. Secondly, the limited number of *RTK* fusions-positive MSI-H CRC patients included in this study, may introduce bias in the comprehensive assessment of molecular and immune characteristics. Thirdly, only one *NTRK* fusion-positive CRC sample was selected to assess the methylation level which might not represent the methylation level of the study cohort. Finally, we only presented a case report of an MSI-H CRC patient harboring *NTRK* fusion, who had a good response to immunotherapy. As no targeted therapy was administrated, we could not exclude the possibility of targeted therapy being able to achieve a durable response in this patient. Due to the lack of information of the responses to immunotherapy from both in-house cohort and TCGA COAD)/READ database, it is difficult for us to validate our findings in the current study. More validations are required in the future.

## Conclusion

Our study characterized MSI-H altered subgroup as a novel subtype of MSI-H CRC patients with unique clinical, molecular and immune features. DEGs and immune-related signatures identified in MSI-H-altered CRC patients revealed the presence of an immune-active microenvironment rather than an immune-suppressive state. Our preliminary findings provided clinical evidence for the use of immunotherapy in MSI-H CRC patients harboring targetable alterations. Based on these results, a prospective multicenter study is ongoing.

## Data availability statement

The raw sequence data reported in this paper have been deposited in the Genome Sequence Archive (Genomics, Proteomics & Bioinformatics 2021) in National Genomics Data Center (Nucleic Acids Res 2022), China National Center for Bioinformation / Beijing Institute of Genomics, Chinese Academy of Sciences (GSA-Human: HRA002868) that are publicly accessible at https://ngdc.cncb.ac.cn/gsa-human.

## Ethics statement

This study was approved by the research ethics committee of the First Affiliated Hospital, Zhejiang University School of Medicine, China (NO. IIT20210185B). The patients/participants provided their written informed consent to participate in this study. Written informed consent was obtained from the individual(s) for the publication of any potentially identifiable images or data included in this article.

## Author contributions

QS, LT, and WJ designed research. HH, WH, NC, YH, and GW performed research. FY, XZ, YL, YD, and WZ contributed new reagents/analytic tools. HH and WJ wrote the paper. All authors contributed to the article and approved the submitted version.
