# Supplementary material for: Genomic and transcriptomic analysis of MSI-H colorectal cancer patients with targetable alterations identifies clinical implications for immunotherapy
Source: Front Immunol. 2023 Jan 9;13:974793. doi: 10.3389/fimmu.2022.974793 (PMC9870311; doi:10.3389/fimmu.2022.974793)
Supplement: Supplementary file 1 [file DataSheet_1.docx]

Supplementary Tables:

Supplementary Table 1: The definition of targetable alterations in this study.

| Fusion (n=16) | *ALK*, *BRAF*, *EGFR*, *ERBB2*, *ERBB4*, *FGFR1/2/3*, *KIT*, *MET*, *NTRK1/2/3*, *RET*, *ROS1* and *NRG1* |
| --- | --- |
| Missense(n=2) | *BRAF^V600E^* and *KRAS^G12C^* |
| Amplification (n=1) | *ERBB2* |

Supplementary Table 2：Clinicopathological characteristics of MSI-H and MSS CRC patients

|  | Overall (n = 218) | MSI-H (n = 126) | MSS (n = 92) | p |
| --- | --- | --- | --- | --- |
| **Age** |  |  |  | 0.739 |
| Mean (SD) | 59.41 (13.78) | 59.98 (13.61) | 58.64 (14.05) |  |
| Median [IQR] | 60.00 [52.00, 70.00] | 59.00 [52.00, 70.00] | 60.00 [51.50, 70.00] |  |
| Missing | 1 ( 0.46%) | 1 ( 0.79%) | 0 ( 0.00%) |  |
| **Gender** |  |  |  | 0.329 |
| female | 87 (39.91%) | 54 (42.86%) | 33 (35.87%) |  |
| male | 131 (60.09%) | 72 (57.14%) | 59 (64.13%) |  |
| **Stage** |  |  |  | < 0.001 |
| I | 9 ( 4.13%) | 8 ( 6.35%) | 1 ( 1.09%) |  |
| II | 67 (30.73%) | 63 (50.00%) | 4 ( 4.35%) |  |
| III | 47 (21.56%) | 36 (28.57%) | 11 (11.96%) |  |
| IV | 95 (43.58%) | 19 (15.08%) | 76 (82.61%) |  |
| **TMB** |  |  |  | < 0.001 |
| Mean(SD) | 49.26 (63.80) | 75.05 (60.65) | 13.93 (49.71) |  |
| median[IQR] | 36.89 [6.51, 66.80] | 58.82 [41.87, 78.26] | 5.56 [2.99, 7.98] |  |
| **Tumor side** |  |  |  | < 0.001 |
| Left | 91 (41.74%) | 29 (23.02%) | 62 (67.39%) |  |
| Right | 79 (36.24%) | 63 (50.00%) | 16 (17.39%) |  |
| Missing | 48 (22.02%) | 34 (26.98%) | 14 (15.22%) |  |

Supplementary Table 3: 83 immune related genes covered by GEP panel

| Gene | Signature | Merck18 |
| --- | --- | --- |
| *ACTA2* | TGFβ | N |
| *COL4A1* | TGFβ | N |
| *TAGLN* | TGFβ | N |
| *SH3PXD2A* | TGFβ | N |
| *TGFB1* | TGFβ | N |
| *TGFBR2* | TGFβ | N |
| *CLDN3* | EMT | N |
| *CLDN7* | EMT | N |
| *CLDN4* | EMT | N |
| *CDH1* | EMT | N |
| *VIM* | EMT | N |
| *TWIST1* | EMT | N |
| *ZEB1* | EMT | N |
| *ZEB2* | EMT | N |
| *FLNA* | EMT | N |
| *EMP3* | EMT | N |
| *CALD1* | EMT | N |
| *FN1* | EMT | N |
| *FOXC2* | EMT | N |
| *LOX* | EMT | N |
| *FBN1* | EMT | N |
| *TNC* | EMT | N |
| *PDCD1* | PD | N |
| *CD274* | PD | Y |
| *AXL* | IPRES_eCD8T | N |
| *ROR2* | IPRES_eCD8T | N |
| *LOXL2* | IPRES_eCD8T | N |
| *VEGFC* | IPRES_eCD8T | N |
| *FLT1* | IPRES_eCD8T | N |
| *ANGPT2* | IPRES_eCD8T | N |
| *IL10* | IPRES_eCD8T | N |
| *TNFRSF9* | IPRES_eCD8T | N |
| *CD200* | IPRES_eCD8T | N |
| *CD28* | IPRES_eCD8T | N |
| *CD40* | IPRES_eCD8T | N |
| *CD80* | IPRES_eCD8T | N |
| *CD86* | IPRES_eCD8T | N |
| *CTLA4* | IPRES_eCD8T | N |
| *TNFRSF14* | IPRES_eCD8T | N |
| *TNFSF4* | IPRES_eCD8T | N |
| *HAVCR2* | IPRES_eCD8T | N |
| *C10orf54* | IPRES_eCD8T | N |
| *STAT3* | IPRES_eCD8T | N |
| *IKBKE* | IPRES_eCD8T | N |
| *CD276* | IPRES_eCD8T | Y |
| *CD8A* | Teff_interferon_gama | Y |
| *GZMA* | Teff_interferon_gama | N |
| *GZMB* | Teff_interferon_gama | N |
| *EOMES* | Teff_interferon_gama | N |
| *CXCL9* | Teff_interferon_gama | Y |
| *CXCL10* | Teff_interferon_gama | N |
| *TBX21* | Teff_interferon_gama | N |
| *IFNG* | Teff_interferon_gama | N |
| *CXCL11* | Teff_interferon_gama | N |
| *PTPRC* | Teff_interferon_gama | N |
| *STAT1* | Teff_interferon_gama | Y |
| *IDO1* | Teff_interferon_gama | Y |
| *CD74* | Teff_interferon_gama | N |
| *CD4* | Teff_interferon_gama | N |
| *CXCR6* | Teff_interferon_gama | Y |
| *CD3D* | Teff_interferon_gama | N |
| *IL2RG* | Teff_interferon_gama | N |
| *IL2RB* | Teff_interferon_gama | N |
| *CD2* | Teff_interferon_gama | N |
| *CCR5* | Teff_interferon_gama | N |
| *CXCL13* | Teff_interferon_gama | N |
| *CD27* | Teff_interferon_gama | Y |
| *PRF1* | Teff_interferon_gama | N |
| *LAG3* | Teff_interferon_gama | Y |
| *CCL5* | Teff_interferon_gama | Y |
| *HLA-DRA* | Teff_interferon_gama | N |
| *CD3E* | Teff_interferon_gama | N |
| *NKG7* | Teff_interferon_gama | Y |
| *HLA-E* | Teff_interferon_gama | Y |
| *CIITA* | Teff_interferon_gama | N |
| *TAGAP* | Teff_interferon_gama | N |
| *GZMK* | Teff_interferon_gama | N |
| *CMKLR1* | Teff_interferon_gama | Y |
| *HLA-DQA1* | Teff_interferon_gama | Y |
| *HLA-DRB1* | Teff_interferon_gama | Y |
| *PDCD1LG2* | Teff_interferon_gama | Y |
| *PSMB10* | Teff_interferon_gama | Y |
| *TIGIT* | Teff_interferon_gama | Y |

Supplementary table 4: The comparisons of P- values before and after the adjustment.

| P-value | Teff IFN-gamma | CYT | MERCK 18 | IPRES | EMT | TGF Beta |
| --- | --- | --- | --- | --- | --- | --- |
| Before (Figure 6B-G) | 0.05 | 0.06 | 0.028 | **<0.05** | 0.15 | 0.16 |
| After adjustment | 0.766 | 0.074 | 0.11 | **0.03** | 0.613 | 0.116 |
