# Supplementary material for: Genomic and transcriptomic analysis of MSI-H colorectal cancer patients with targetable alterations identifies clinical implications for immunotherapy
Source: Front Immunol. 2023 Jan 9;13:974793. doi: 10.3389/fimmu.2022.974793 (PMC9870311; doi:10.3389/fimmu.2022.974793)

Supplementary Figures:

Supplementary Figure1: Oncoprints of MSI-H and MSS CRC patients included in this study.

Supplementary Figure 2: The prevalence of major targetable alterations in MSI-H and MSS groups. A. in-house cohort; B. TCGA-COAD/READ database *p < 0.05, **p < 0.01, ****p< 0.0001.


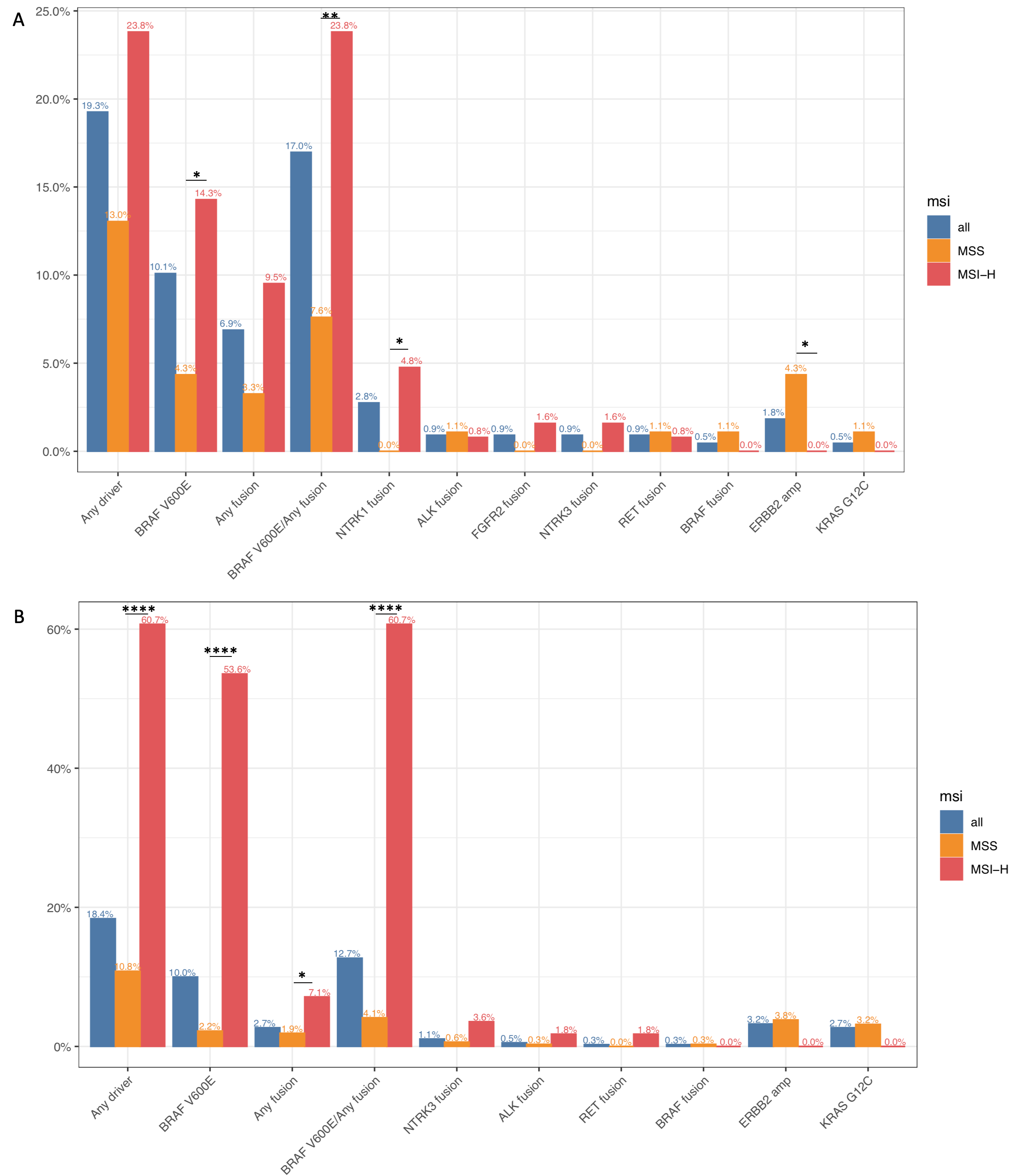


Supplementary Figure 3: Gene set enrichment analysis (GSEA) of the signaling pathways significantly enriched in the MSI-H altered group. A. colorectal cancer-related pathway; B. WNT signaling pathway; C. VEGF signaling pathway; D. TGF_Beta signaling pathway.

Supplementary Figure 4: The detailed comparison of the ICIs biomarker TMB and TNB in MSI-H altered and wt CRC subgroups. A - B. Comparison of TMB levels between the MSI-H altered and wt subgroups. C - D. Comparison of TNB levels between the MSI-H altered and wt subgroups.


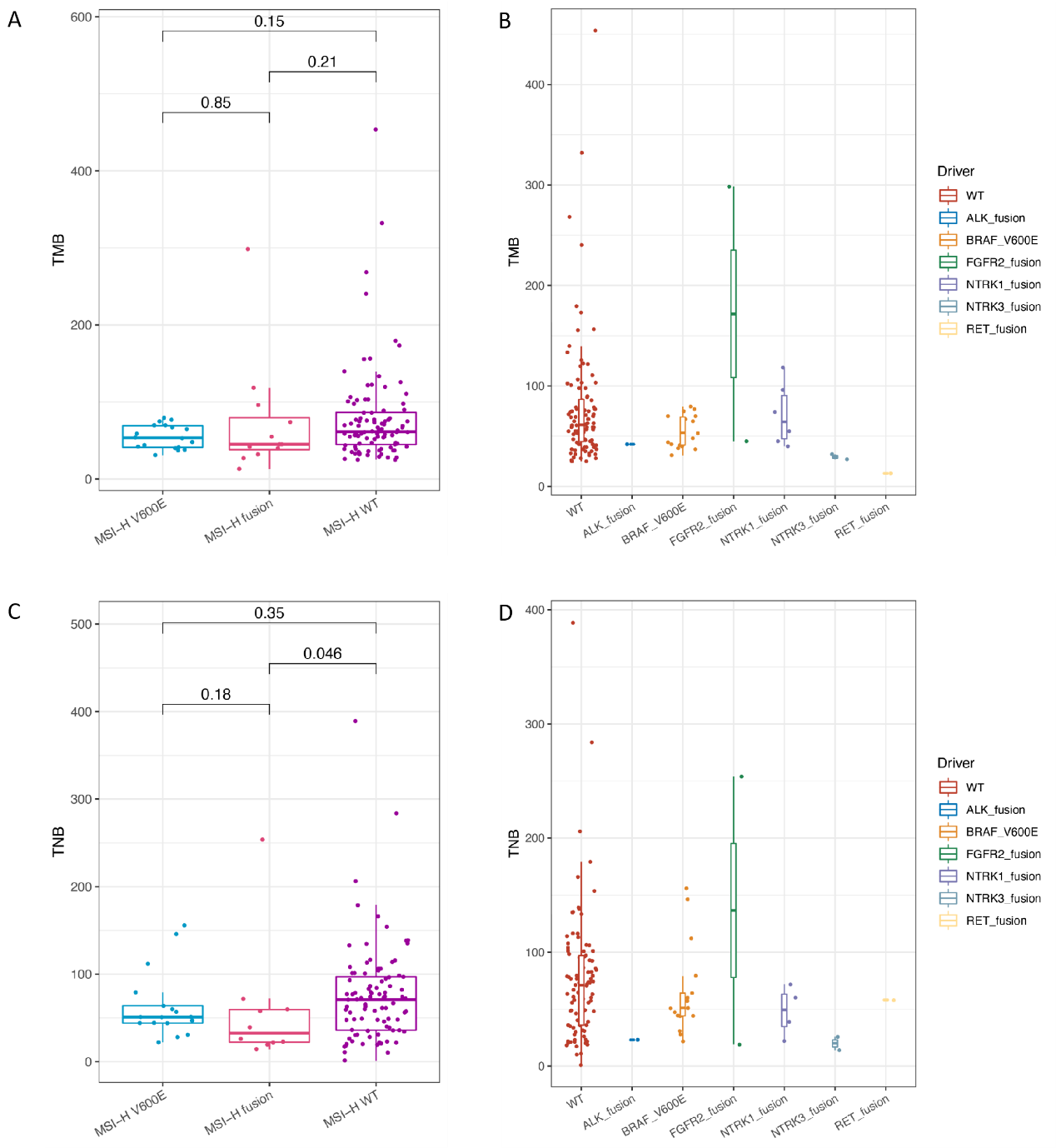


Supplementary Figure 5: Comparison of immune-related genes and immune-related scores in MSI-H altered and wt CRC subgroups. A. Comparison of immune-related genes between the MSI-H altered and wt subgroups. B. Comparison of immune-related scores between the MSI-H altered and wt subgroups.


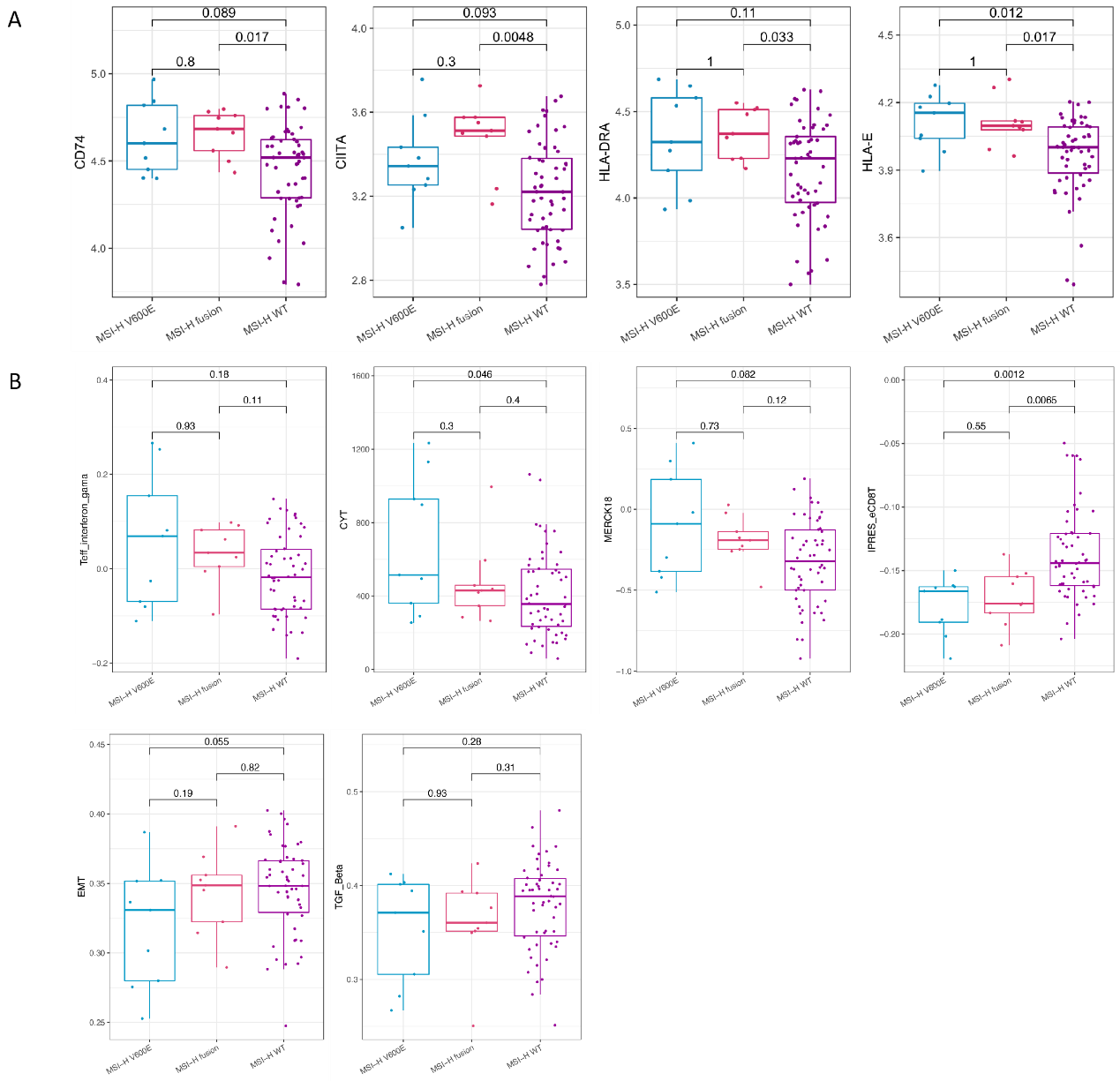


Supplementary Figure 5:

Supplementary Figure 6: The IGV image representing the *TPM3-NTRK1* fusion (T9:N9) in the colon surgical sample. IGV, Integrative Genomics Viewer.


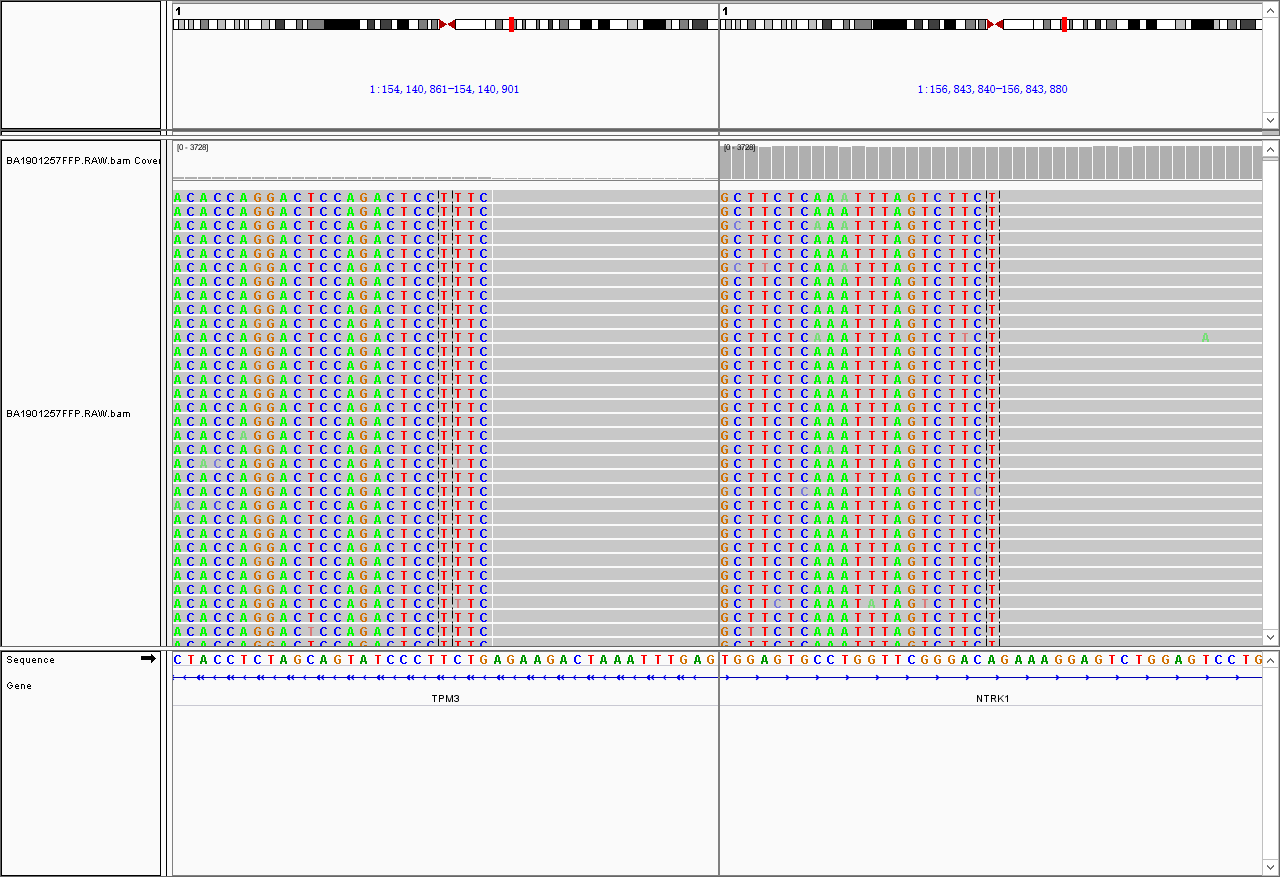

Supplement: Supplementary file 2 [file DataSheet_2.docx]
